# Effectiveness of corticosteroids versus adrenocorticotropic hormone for infantile spasms: a systematic review and meta‐analysis

**DOI:** 10.1002/acn3.50922

**Published:** 2019-10-27

**Authors:** Yin‐Hsi Chang, Chiehfeng Chen, Shu‐Huey Chen, Yu‐Chun Shen, Yung‐Ting Kuo

**Affiliations:** ^1^ School of Medicine College of Medicine Taipei Medical University Taipei Taiwan; ^2^ Department of Public Health School of Medicine College of Medicine Taipei Medical University Taipei Taiwan; ^3^ Division of Plastic Surgery Department of Surgery Wan Fang Hospital Taipei Medical University Taipei Taiwan; ^4^ Cochrane Taiwan Taipei Medical University Taipei Taiwan; ^5^ Department of Pediatrics School of Medicine College of Medicine Taipei Medical University Taipei Taiwan; ^6^ Department of Pediatrics Shuang Ho Hospital, Ministry of Health and Welfare Taipei Medical University New Taipei City Taiwan; ^7^ Taipei Cancer Center Taipei Medical University Taipei Taiwan

## Abstract

**Objective:**

To compare the therapeutic effectiveness of oral corticosteroids with that of adrenocorticotrophic hormone for infantile spasms.

**Methods:**

PubMed, Embase, Scopus, and the Cochrane library were searched to retrieve studies published before December 2018 to identify pediatric patients with a diagnosis of infantile spasms. The interventions of oral corticosteroids and adrenocorticotrophic hormone were compared. We included only randomized controlled trials that reported the cessation of spasms as treatment response. The primary outcome was clinical spasm cessation on day 13 or 14. The secondary outcomes were the resolution of hypsarrhythmia, side effects, continued spasm control, spasm relapse rate, and subsequent epilepsy rate. Following the Preferred Reporting Items for Systematic Reviews and Meta‐Analyses, the study‐level quality assessment was conducted using the Cochrane risk‐of‐bias tool.

**Results:**

After extensive review, 39 articles were included for meticulous evaluation. Five randomized controlled trials with a total of 239 individuals were eligible for further analysis. No significant difference was detected between the corticosteroids and adrenocorticotrophic hormone in the cessation of clinical spasms (odds ratio [OR]: 0.54; 95% confidence interval [CI]: 0.16 to 1.81; *P* = 0.32). The subgroups of high‐dose prednisolone versus adrenocorticotrophic hormone and low‐dose prednisone versus adrenocorticotrophic hormone also exhibited no significant difference. Furthermore, the two subgroups did not differ in terms of hypsarrhythmia resolution, side effects, relapse rate, or subsequent epilepsy rate.

**Interpretation:**

This meta‐analysis suggests that high‐dose prednisolone is not inferior to adrenocorticotrophic hormone and that it be considered a safe and effective alternative treatment.

## Introduction

Infantile spasms (IS), or West syndrome, is an epileptic syndrome characterized by epileptic spasms, hypsarrhythmia on electroencephalography (EEG), and high risk of neurodevelopmental regression. It occurs mainly between the ages of 3 and 12 months with an incidence of 0.25 to 0.43 per 1000 live births and a peak age of onset between 4 and 7 months.^1^, ^2^ Numerous etiologies including structural, genetic, metabolic, and perinatal causes have been linked to IS.^3^, ^4^

Cessation of spasms is the major outcome measure for this disease. EEG serves as a useful tool for evaluation. It has been demonstrated that the neurodevelopmental outcome can be significantly improved if the spasms are controlled early.^5^ Delayed treatment can lead to a worse prognosis including psychomotor regression and other types of seizure in later childhood and adult life.^6^, ^7^

Treatments vary including antiepileptic drugs, corticotropic hormone, pyridoxine, and a ketogenic diet.^8^, ^9^, ^10^ IS is difficult to control with conventional antiepileptic drugs.^11^ Vigabatrin is recommended specifically for treatment in IS secondary to tuberous sclerosis complex (TSC).^9^, ^12^ The most widely accepted effective treatment for IS not secondary to TSC is hormone therapy.^13^ It is hypothesized that an excess of corticotrophin‐releasing hormone (CRH) enhances the excitant effects of CRH for numerous neurons. Furthermore, the CRH receptors are especially abundant in the early neonatal period.^14^ Theoretically, adrenocorticotropic hormone (ACTH) plays a major role in treating IS by suppressing endogenous CRH through a negative feedback pathway. ACTH is preferred as the first‐line treatment in most patients;^8^ however, it is expensive and difficult to obtain in some countries.^15^, ^16^ As a result, the corticosteroids, prednisone, prednisolone, and methylprednisolone, have been used effectively off‐label for decades.^12^, ^17^, ^18^ The United Kingdom Infantile Spasms Study (UKISS) revealed no significant differences in treatment outcomes between intramuscular synthetic ACTH (tetracosactide) and oral prednisolone in subgroup analysis.^19^ Patients and their families can benefit from proof that corticosteroids are an alternative treatment because they are less costly and easier to obtain and administer than ACTH.

To the best of our knowledge, no meta‐analysis in the literature has directly compared prednisolone with ACTH. Our meta‐analysis compared the effectiveness and safety of ACTH to two oral corticosteroids, prednisone and prednisolone.

## Methods

### Search strategy and selection criteria

This study was conducted and reported in accordance with the Preferred Reporting Items for Systematic Reviews and Meta‐Analyses. The electronic databases PubMed, Embase, Scopus, and the Cochrane Central Register of Controlled Trials were searched using the terms (“infantile spasms” OR “West syndrome” OR “epileptic spasm”) AND (“adrenocorticotropic hormone” OR “ACTH”) AND (“steroids” OR “corticosteroids” OR “prednisolone”). The databases were searched for dates before December 2018. The inclusion criteria were as follows: (I) pediatric patients less than 5 years old, (II) diagnosis of IS or West syndrome based on both clinical spasms and EEG findings, (III) comparison of oral corticosteroids with intramuscular ACTH, (IV) cessation of spasms reported as the treatment response, and (V) randomized controlled trials (RCTs) published as full‐text trials irrespective of the language. Studies were excluded if they were not randomized. Conference abstracts, reviews, editorials, letters, and unpublished studies were also excluded.

### Data extraction and study quality assessment

Two reviewers (YHC, YTK) independently screened the titles and abstracts of studies to identify trials meeting the inclusion criteria. When disagreements arose between reviewers, the full text of the paper was retrieved, and the disagreements were reconsidered and discussed until a consensus was reached. We adopted the Cochrane risk‐of‐bias tool to perform risk‐of‐bias assessment.^20^ Domains included the adequacy of the randomization, allocation concealment, blinding of patients, and outcome assessors. Incomplete outcome data and selective outcome reporting were assessed. Publication bias was assessed by checking for asymmetry in funnel plots if at least 10 studies were included.

### Outcome and data analysis

The primary outcome measure for this analysis was the cessation of clinical spasms for at least 48 h after oral corticosteroids or intramuscular ACTH had been administered. The outcome was measured as a dichotomous variable on day 14 and as a continuous variable of time until spasm cessation (measured in days) for all days through day 14. Secondary outcomes were the resolution of hypsarrhythmia on EEG, common side effects related to the treatments, continued spasm control through 1 year of follow‐up, spasm relapse rate, and subsequent epilepsy rate. Relapse was defined as a single spasm or one or more clusters of spasms in a child who previously had appeared free of spasms within the study period. Subsequent epilepsy was defined as any seizure type other than IS after the commencement of therapy. A random‐effects model was employed to pool individual odds ratios (ORs) for the dichotomous outcomes and standardized mean differences for the continuous outcomes. Statistical significance was defined as *P* < 0.05. To assess the significance of the between‐study heterogeneity, the Q statistic was calculated. The magnitude of the between‐study heterogeneity was determined through I^2^ tests; values> 50% were regarded as considerable heterogeneity. All analyses were performed using the Review Manager software package, version 5.3 (Cochrane Collaboration, Copenhagen, Denmark). The results of the meta‐analysis are presented as forest graphs.

## Results

### Literature search and selection

**Figure 1:**
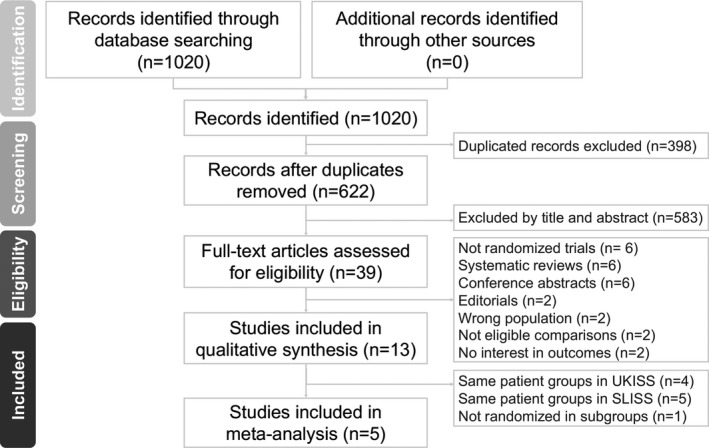
Flow diagram of the search process and search results.

From the 1020 initial search results, we retrieved 622 nonduplicate studies for a review of their titles and abstracts and included 39 articles for meticulous evaluation after eliminating ineligible patients and interventions. We further excluded six nonrandomized studies, six systematic reviews without raw data, six conference abstracts, two editorials, and six other articles that did not meet the inclusion criteria. In the remaining published 13 articles, one study group had four articles reporting related data;^7^, ^19^, ^21^, ^22^ another study group had five articles with related data.^23^, ^24^, ^25^, ^26^, ^27^ Thus, to avoid using overlapping data, we selected the most recently published report for each of these groups to be included in the analyses; this also provided the outcomes with the longest follow‐up. One extensive randomized, multicenter, open‐label trial comparing hormonal treatment and hormonal treatment with vigabatrin did not provide enough data for us to analyze the outcomes of the prednisolone and ACTH subgroups. Finally, five remaining RCTs met our criteria and were included in this meta‐analysis (Fig. 1).

### Study characteristics and study quality

**Figure 2:**
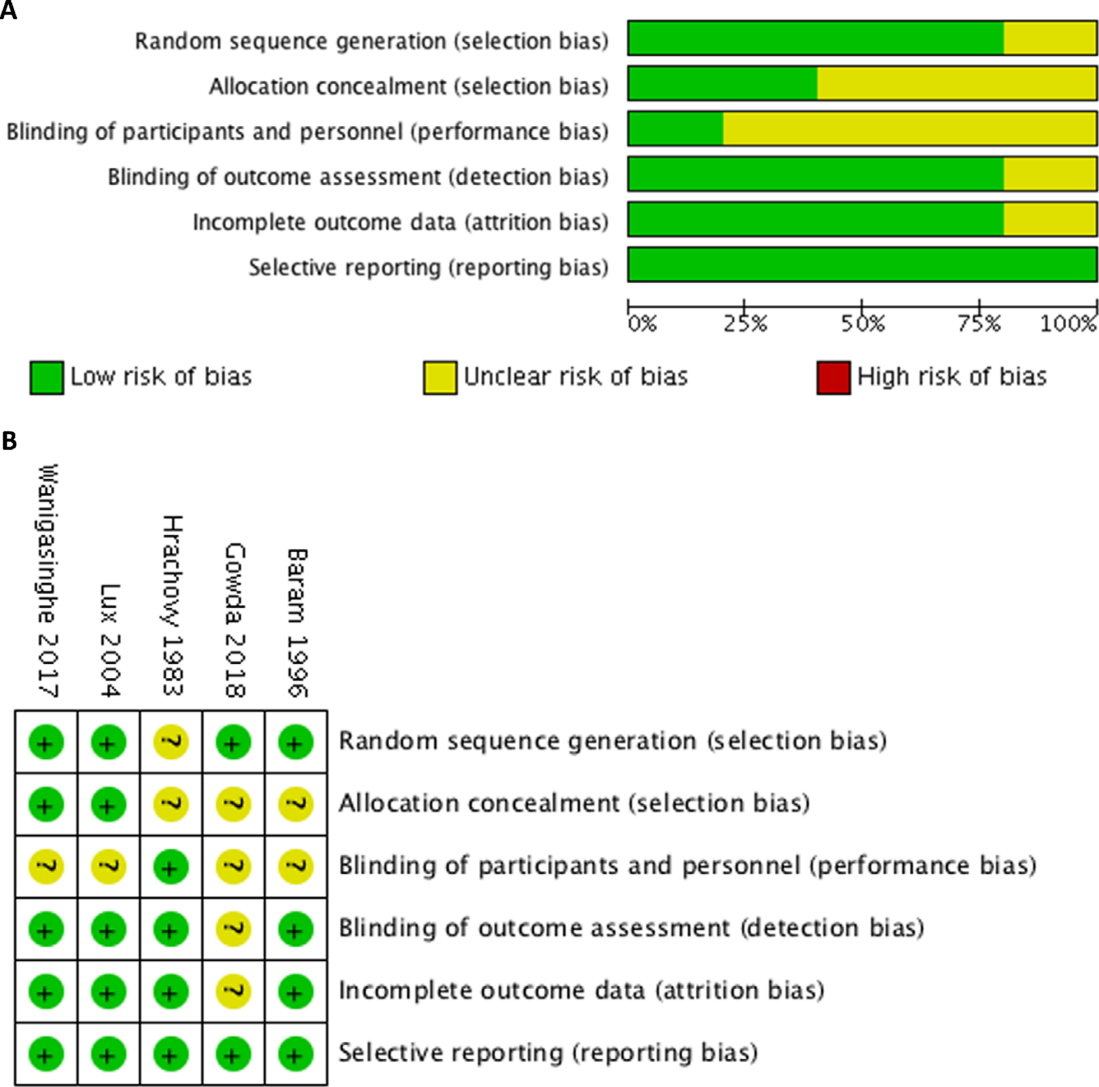
Methodological quality. (A) Risk‐of‐bias summary of the randomized controlled trial. (B) Risk‐of‐bias graph of the randomized controlled trials.

The five RCTs in the final quantitative analysis included 239 participants. Patients were mainly aged from 2 months to 2 years. One study included patients up to 5 years old.^28^ IS was consistently diagnosed on the basis of clinical presentation and EEG studies. Three studies excluded patients with a history of tuberous sclerosis and four excluded those who had already received other treatments.^19^, ^27^, ^28^, ^29^ Variable lead time to treatment, the time between the onset of spasms and the commencement of treatment were noted in Hrachovy’s study.^30^ Hrachovy *et al.* and Baram *et al.* used the porcine ACTH in their studies.^29^, ^30^ Lux *et al.*, Wanigasinghe *et al.*, and Gowda *et al.* administered synthetic ACTH.^19^, ^27^, ^28^ The characteristics of all five studies and administration details of each therapy are listed in Table 1. The final results of the risk‐of‐bias assessment are presented in Figure 2; each RCT was categorized on the basis of bias potential as high risk, low risk, or unclear.

**Table 1:** Characteristics of the included trials. Intervention was designed on a 2‐week basis.

Author year	No. of Patients	Mean age (range), month	Intervention		Outcome
			Corticosteroid	ACTH	
Gowda 2019	34	11.5 (2–60)	Prednisolone 4 mg/kg per d (max 60 mg/d)	100 U/BSA	Primary: spasm cessation, time taken for cessation Secondary: S/E, relapse rate, subsequent epilepsy rate, F/U at 3, 6 months
Wanigasinghe 2017	97	9.1 (2–24)	Prednisolone 40–60 mg/d in 4 divided doses	40–60 IU/every other day	Primary: spasm cessation, EEG remission Secondary: days taken to remission, quantitative reduction of spasms, S/E, relapse rates F/U at 6 wks and 3, 6, 12 months
Lux 2005	107	6 (2–12)	Prednisolone 40–60 mg/d in 4 divided doses	40–60 IU/ every other day	Primary: spasm control Secondary: S/E, relapse rates, development VABS, F/U at 14 months
Baram 1996	29	6.3 (2–21)	Prednisone 2 mg/kg per d in 2 divided doses	150 U/m^2^ per day in 2 divided doses	Spasms cessation, EEG resolution, time for response, S/E, relapse rates, subsequent epilepsy rate
Hrachovy 1983	24	NS (3.5–24)	Prednisone 2 mg/kg per d	20 IU/day	Spasms cessation, EEG resolution, S/E relapse rates, developmental delay F/U at 6 wks

Abbreviations: No., number; NS, not stated; max, maximum; BSA, body surface area; kg, kilogram; d, day; wks, weeks; F/U, follow‐up; S/E, side effects; VABS, Vineland adaptive behavior scales.

### Primary outcome: cessation of clinical spasms

**Figure 3:**
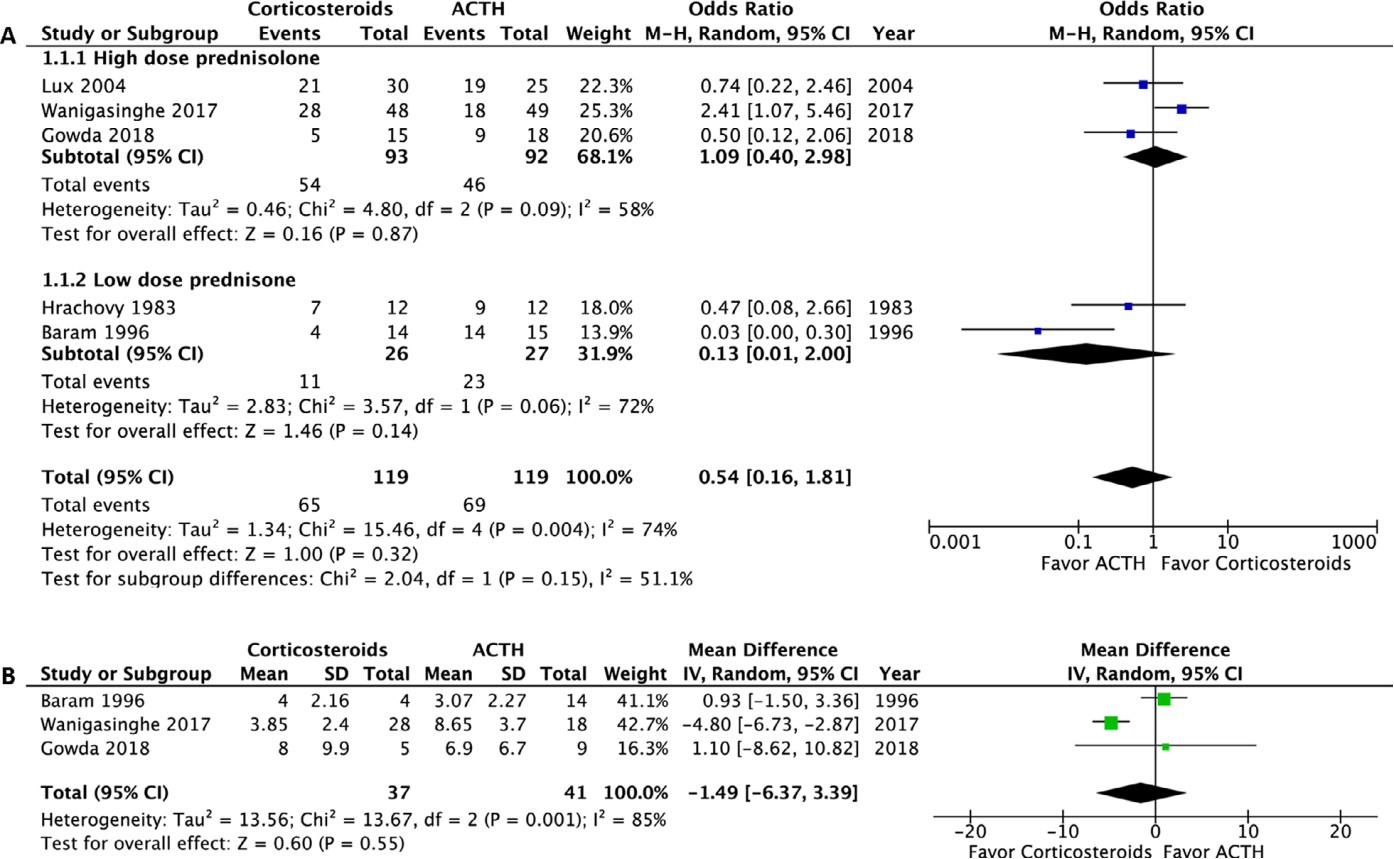
Forest plot of the primary outcome. (A) The cessation rate of clinical spasms. (B) The time until spasm cessation (days).

The primary outcome was assessed in 238 of the 239 participants. One patient was lost to follow‐up after 14 days of treatment.^28^ Analysis of the primary outcome revealed no significant difference in overall spasm cessation (OR: 0.54; 95% confidence interval [CI]: 0.16 to 1.81; *P* = 0.32). In the subgroup analyses, no significant difference in primary outcome was observed between high‐dose prednisolone and ACTH (OR: 1.01; 95% CI: 0.40 to 2.98; *P* = 0.87) or between low‐dose prednisone and ACTH (OR: 0.13; 95% CI: 0.01 to 2.00; *P* = 0.14) (Fig. 3A). The magnitude of heterogeneity in the high‐dose prednisolone group (*I* ^2^ = 0.58) was lower than in the low‐dose prednisone group (*I* ^2^ = 0.72). Three of the five RCTs studied the time until response; no significant difference was observed (mean difference −1.49, 95% CI: −6.37 to 3.39; *P* = 0.55).^27^, ^28^, ^29^ The mean time to spasm cessation ranged from 3 to 8 days (Fig. 3B).

### Secondary outcomes

**Figure 4:**
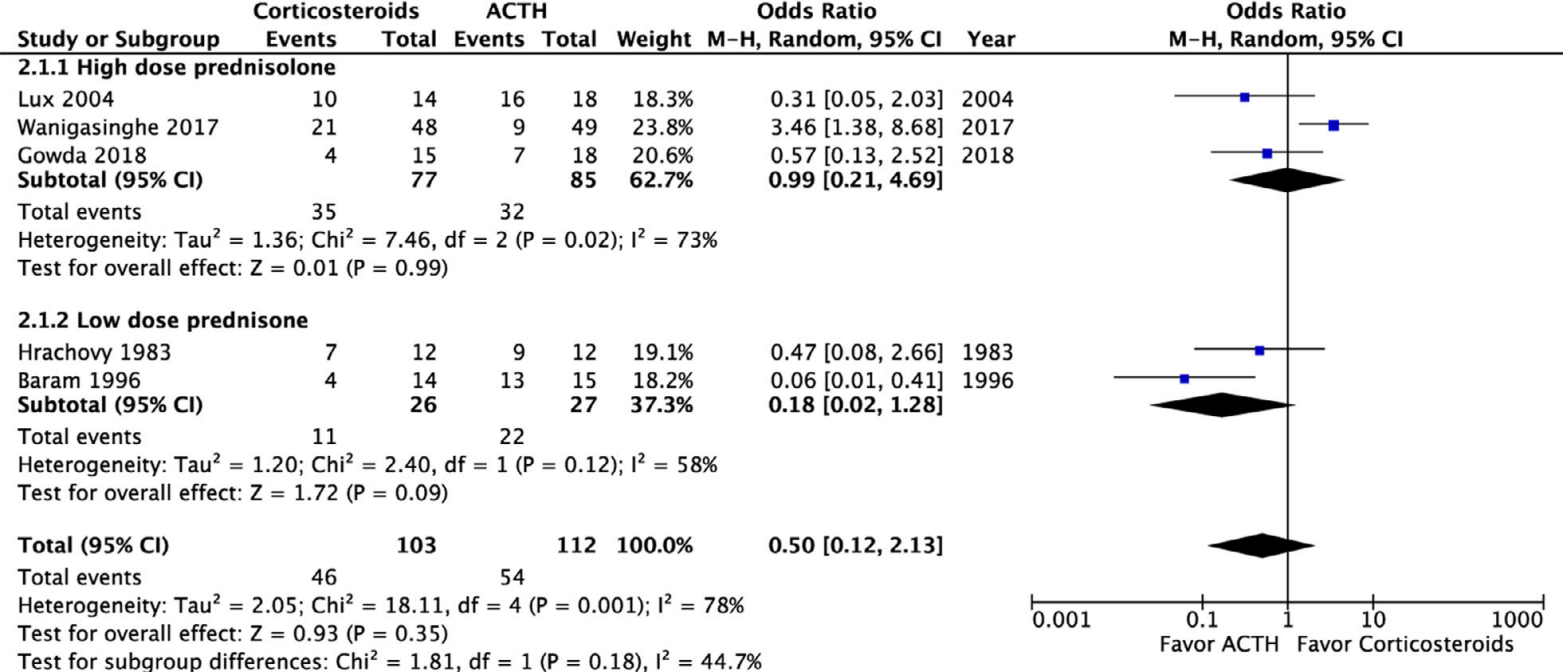
Forest plot of hypsarrhythmia resolution rate on EEG.

**Figure 5:**
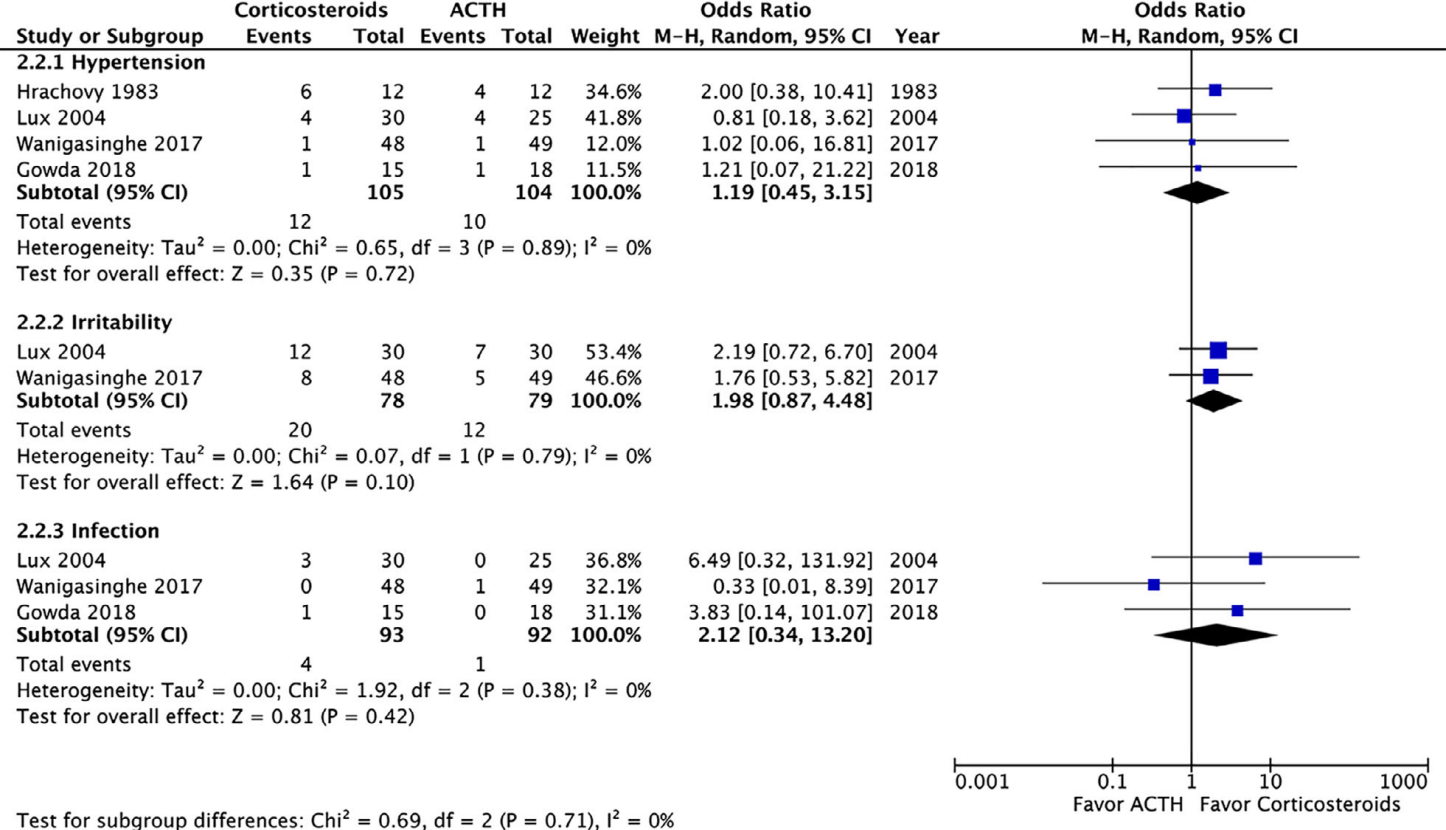
Forest plot of the common side effects.

**Figure 6:**
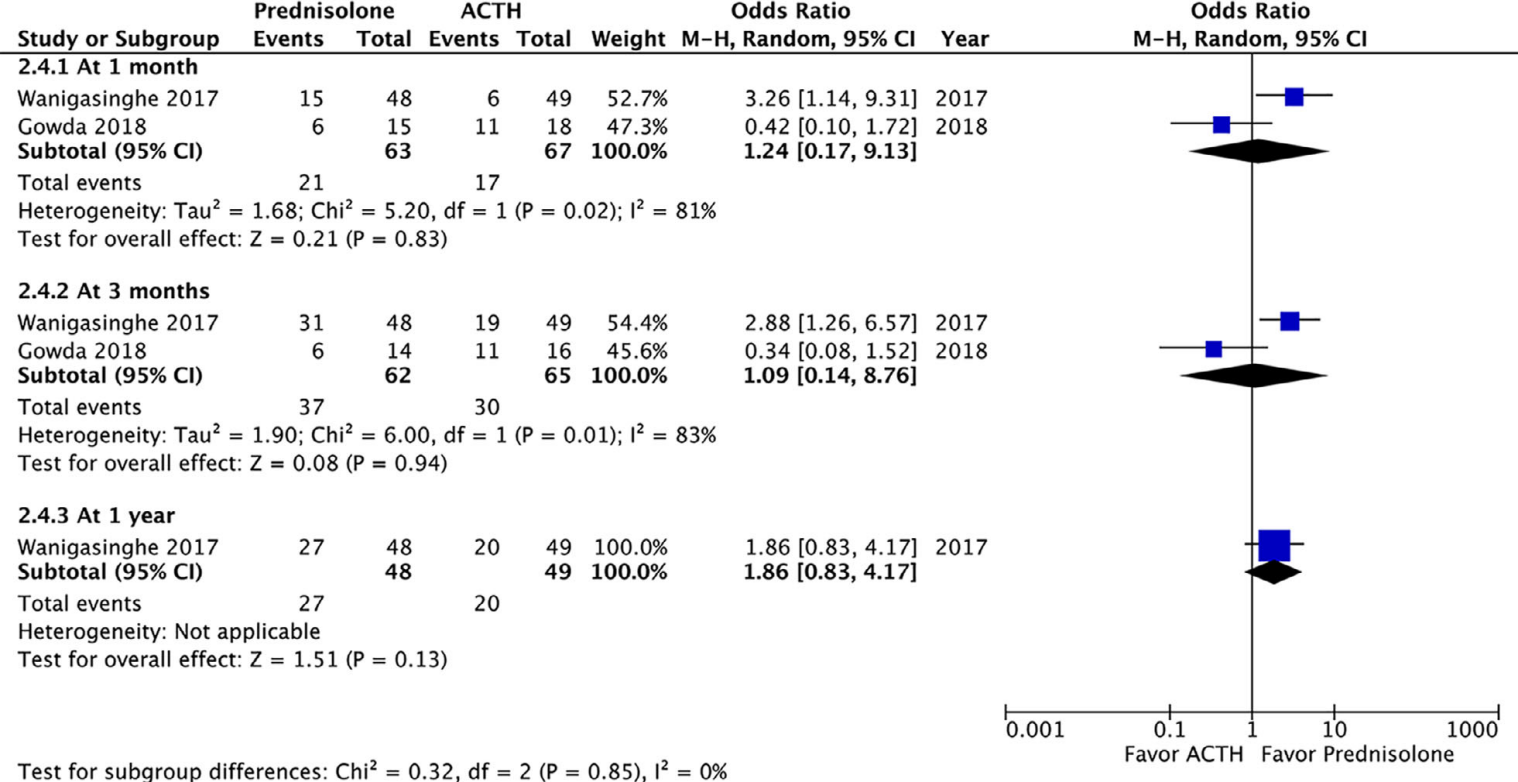
Forest plot of continued spasm control through up to 1 year of follow‐up.

**Figure 7:**
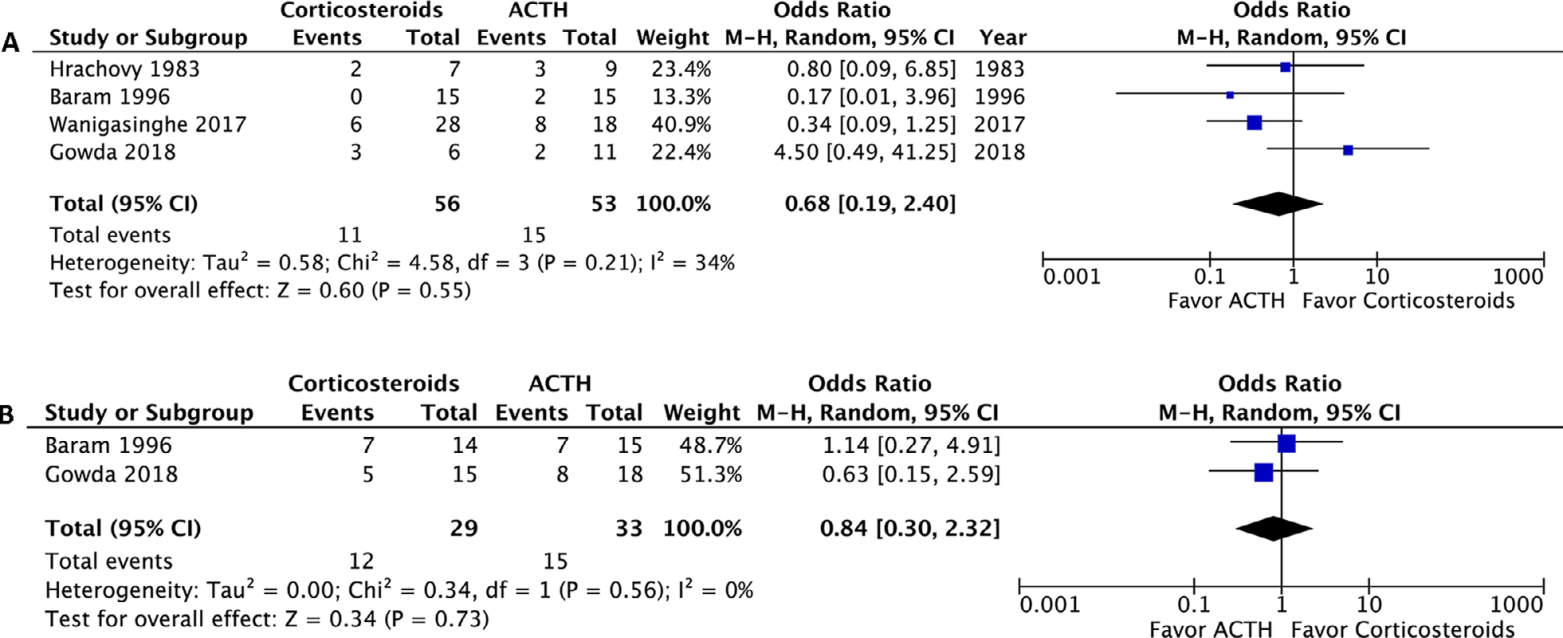
(A) Forest plot of the relapse rate. (B) Forest plot of the subsequent epilepsy rate.

A post‐treatment EEG was performed on 215 patients and revealed no significant difference in the resolution of hypsarrhythmia between the corticosteroid and ACTH groups (OR: 0.5; 95% CI: 0.12 to 2.13; *P* = 0.35). Moreover no significant difference was observed between high‐dose prednisolone and ACTH (OR: 0.99; 95% CI: 0.21 to 4.69; *P* = 0.99) or between low‐dose prednisone and ACTH (OR: 0.18; 95% CI: 0.02 to 1.28; *P* = 0.09) (Fig. 4). We compared the side effects listed by four studies and analyzed those of clinical concern.^21^, ^27^, ^28^, ^30^ The rates of hypertension (OR: 1.35; 95% CI: 0.46 to 3.98; *P* = 0.59), irritability (OR: 1.98; 95% CI: 0.87 to 4.48; *P* = 0.10), and infection (OR: 2.12; 95% CI: 0.34 to 13.20; *P* = 0.42) were not significantly different between the two groups (Fig. 5). In total, two patients in the prednisolone group and one in the ACTH group stopped the therapy because of side effects.^21^, ^23^ At the 1‐month, 3‐month, and 1‐year follow‐ups, no significant difference was observed in any of the groups (Fig. 6). Furthermore, the relapse rate was not different between the two groups. (OR: 0.68; 95% CI: 0.19 to 2.40; *P* = 0.55) (Fig. 7A). Only two articles addressed subsequent epilepsy; no significant difference was found for this outcome either (OR: 0.84; 95% CI: 0.30 to 2.32; *P* = 0.73) (Fig. 7B).^28^, ^29^

### Assessment of bias

The assessment of the risk of bias in each included RCT is presented in Figure 2. Blinding of the patients and their families is difficult to evaluate because the two drugs were administered through different routes as the patients and their families could tell the difference between the oral and intramuscular route while receiving the therapy. This fact did not affect the efficacy of administration, however. The performance bias of immediate spasm cessation as the primary outcome measure was low because blinding did not affect the clinical spasms. Attrition bias was observed in the analysis of intermediate‐ to long‐term follow‐up in three studies.^19^, ^27^, ^28^ Twelve patients died, and 17 were lost to follow‐up and therefore excluded from the studies. Most of the deaths were related to underlying or systemic diseases. Because publication bias and interstudy heterogeneity might have been present, we conducted subgroup analyses; the results are presented in Figures 2 and 3.

## Discussion

### Key findings

The evidence from this meta‐analysis reveals the effectiveness of oral corticosteroids for treatment of IS. Differences between corticosteroid and ACTH were nonsignificant for all endpoints including the cessation of spasms, resolution of hypsarrhythmia on EEG presentation, relapse rate, and subsequent epilepsy. Furthermore, the patients in the corticosteroid group did not have significantly more severe side effects. Thus, the results of this meta‐analysis suggest that corticosteroid is not inferior to ACTH and that it can be considered a safe and effective alternative treatment.

### Comparison with other studies

To the best of our knowledge, the present work is the first meta‐analysis of RCTs that directly compare corticosteroids with ACTH in the treatment of IS. Although steroids have been used to treat IS since 1958,^31^ the first randomized study using corticosteroids was published in 1983.^30^ Subsequently, the UKISS revealed similar response rates between ACTH and prednisolone.^19^, ^21^ Extensive research has been conducted in the past 15 years, but the evidence has not been sufficiently strong enough establish the use of corticosteroids as an alternative to ACTH. Because of this, ACTH is the preferred medical treatment according to previous guidelines.^8^, ^32^ A commonly used ACTH dose is 40 IU/day for 2 weeks, and it may be increased to 80 IU/day.^33^ A 2012 systematic review evaluated the role of corticosteroids in treating IS and concluded that their efficacy is similar to that of low‐dose ACTH and inferior to that of high‐dose ACTH.^18^ More recent evidence‐based publications, including a Cochrane review, have indicated that hormone therapy (ACTH and prednisolone) should be the first‐line treatment option.^9^, ^12^ In a systematic review, Song et al. suggested that prednisolone is effective and tolerable.^10^

The growing evidence of the effectiveness of prednisolone has likely contributed to the use of high‐dose prednisolone in the subsequent studies. High‐dose prednisolone (4 mg/kg per day) has now been proven to be more effective than low‐dose prednisolone (2 mg/kg per day).^34^ Our study performed a more comprehensive database review, included new RCTs with 238 total patients, and further supported this conclusion.

The probable mechanism to explain the effectiveness of prednisolone is the suppressive effect on the proepileptogenic neuropeptide CRH.^35^ We know that ACTH promotes the release of glucocorticoids, and glucocorticoids cross the blood–brain barrier (BBB) binding to receptors that are widely expressed throughout the brain. The hormone–receptor complex subsequently modulates the expression of a number of neurotransmitter and neuromodulator genes and, most importantly, the proconvulsant neuropeptide CRH gene in the limbic system.^36^ Most ACTH effects on the central nervous system have been attributed to the activation of glucocorticoid receptors. Brunson *et al*, found that if the ACTH fragments did not release glucocorticoids, they were not effective for treating IS.^36^ This supports the observation that prednisolone by itself is effective in a large proportion of infants with IS. Prednisolone also plays a role in anti‐inflammation and immune regulation. Perhaps a high concentration of prednisolone passes the BBB more efficiently than a low concentration does and thus produces these effects in a more pronounced manner. It remains poorly established whether prednisone and prednisolone are equally effective. Prednisone requires metabolism to convert it into the active form, prednisolone; however, newborn infants have poor HSD11B1 capacity that may be insufficient to convert prednisone to prednisolone. This ability slowly increases only over the first 6 months of life and it is likely that prednisone is not as effective as prednisolone in the treatment of IS in young infants.^9^ To examine this presumption, we performed a subgroup analysis between prednisone and prednisolone in our meta‐analysis. A trend showing less favorable response to prednisone compared to ACTH did not reach statistical significance; however, prednisolone was significantly inferior to ACTH in one study.^29^ A head‐to‐head comparative trial would be helpful to establish a more definite result.

Additional therapies have been reported to treat IS. Vigabatrin is an antiepileptic drug that irreversibly inhibits the breakdown of γ‐aminobutyric acid (GABA) and increases GABA levels in the cerebrospinal fluid.^37^ It has proven more effective in patients with a tuberous sclerosis complex (TSC) when epileptogenesis may be caused by the deficiency of GABA receptors and GABAergic interneurons.^9^ Vigabatrin acts during this step to ameliorate infantile spasms.^38^, ^39^ A recent study of the combination of hormone therapy (prednisolone or ACTH) and vigabatrin demonstrated a significantly superior ability to stop IS within a four‐week period compared to those treated with hormone alone.^40^ Although combination therapy did not reveal better neurodevelopmental of subsequent epilepsy outcomes at 18 months, longer‐term follow‐up of these children at 42 months is ongoing.^41^ Additionally, intravenous methylprednisolone pulse therapy appeared effective in a small trial.^42^ Ganaxolone, a neurosteroid GABA_A_ agonist, is an emerging neonatal anticonvulsant, which may benefit patients with IS.^43^, ^44^ In the future, the optimal dosage route, and combination regimen of hormone therapy must be determined through clinical trials.

### Clinical implications

The benefit of using oral prednisolone is appealing. It is considerably less expensive and more accessible than ACTH, allowing more patients to initiate therapy as early as possible. The time between the onset of spasms and the commencement of treatment, or the lead time to treat, affects the long‐term neurodevelopment outcome.^7^ A longer lead time has been associated with lower Vineland Adaptive Behavior Scales scores.^41^ Because prednisolone is easy to obtain and apply, it minimizes the lag in time to treatment in infants. In addition, the cost of oral prednisolone is approximately US $200 for a month supply, whereas ACTH costs $70,000 per month.^45^ Some families may be unable to afford ACTH even if it is available in their countries. Some developing countries have already been using oral prednisolone for years.^15^ In addition, parents tend to select oral prednisolone for their children instead of intramuscular injection when given the choice.^40^ Our meta‐analysis provides a strong basis for these practice.

The likelihood of developing side effects is another concern when selecting a therapy. The most documented side effects of hormone therapy are hypertension, irritability, gastritis, infections, cushingoid features, and increased appetite.^18^ Sometimes, ACTH may cause several life‐threatening complications including hypertension, cardiomyopathy, and heart failure; only hypertension was observed in our study.^46^ Dosage and exposure time affect toxicity. The side effects of infections, cushingoid features, increased appetite, and weight gain were more commonly noted in the high‐dose prednisolone group than in the low‐dose group, a finding not statistically significant, however.^47^ Another concern is the adrenal suppression associated with using high‐dose prednisolone;^48^ however, Kossoff *et al.* reported fewer adverse effects with prednisolone than ACTH.^45^ Few participants withdrew from the treatment protocol because of intolerable toxicity. Most observed adverse events were not life threatening and typically resolved after the discontinuation of medication.^49^ By keeping the dose as low as possible and the duration as short as possible while maintaining efficacy safe therapy with the least toxicity can be achieved.^33^

The determination of successful treatment of IS usually involves both the cessation of spasms and the resolution of hypsarrhythmia on follow‐up EEG.^50^ Confirming hypsarrhythmia resolution requires experienced pediatric neurologists to interpret the EEG. The typical hypsarrhythmia EEG pattern is a disorganized background with slow high‐amplitude, asynchronous waves and multifocal spike activity.^51^ Hormone therapy has been demonstrated to improve hypsarrhythmia scores, including the background organization, background slowing, amplitude, and spike index.^23^ It also has induced a significant decrease in the theta wave power on EEG.^52^ Focal discharges or normal EEG often appear after the disappearance of hypsarrhythmia on EEG.^11^

The prognosis of IS mainly depends on the response to treatment and the underlying etiology.^5^, ^7^, ^40^ The cessation of spasms after initial treatment resulted in favorable developmental outcomes.^53^ Infants with IS with a known etiology generally have a poor prognosis when the causes are structural, metabolic, or genetic.^54^ Therefore, the management of IS should include determination of the etiology because this can help determine the likelihood of response to treatment, guide therapeutic decisions, and provide a more definitive prognosis for the child.

### Limitations

Our meta‐analysis has some limitations. First, the sample size was small. It was difficult to include more infants or children with IS because retrospective cohorts or observational studies did not meet our inclusion criteria. Some prospective studies lacked randomization of the prednisolone and ACTH groups. Randomized controlled trials give high levels of evidence, but are sometimes not feasible due to cost concerns, ability to enroll, and time. Thus, a more comprehensive meta‐analysis with a larger number of patients can be carried out when more randomized trials are available in the future. Second, we noted heterogeneity in the treatment protocol dosages. Spasm cessation and the incidence or severity of side effects may be related to the dosage. Thus, the results must be interpreted with caution. Subgroup analysis was made between high‐dose prednisolone and low‐dose prednisone for the primary outcome. The heterogeneity in the high‐dose group was lower compared to the overall heterogeneity, but that of the low‐dose prednisone group was also considerable. Other factors might contribute to the heterogeneity such as age difference across studies or the percentage of drop‐out. Age stratification was not possible unless the original age data were provided; however, outcome analysis by age may well be valuable if possible. Third, the long‐term outcome assessment was insufficient because of short follow‐up periods and a lack of standard assessment tools for neurodevelopment. Furthermore, we could only compare the relapse rate and subsequent epilepsy rate retrieved from two studies and these data may not reflect the whole study group.^28^, ^29^ Despite these limitations, the present study provides valuable evidence suggesting the use of prednisolone as an alternative to ACTH for IS.

## Conclusion

This systematic review and meta‐analysis demonstrated that oral prednisolone is as effective as ACTH and the side effects are generally tolerable. It is reasonable to select prednisolone as an alternative to ACTH for treatment of IS.

## Author Contributions

YHC and YTK were the chief investigators and initiated this study. YHC and YTK independently searched the databases, screened the articles, and collected the data. CC and YTK were responsible for the statistical analysis and interpretation of the data. YHC wrote the first draft of the paper. CC and YTK revised the draft for intellectual content. SHC and YCS provided professional advice. All authors contributed to and approved the final version.

## Conflicts of Interest

The authors declare no competing interests.
